# Early Effect of Supplemented Infant Formulae on Intestinal Biomarkers and Microbiota: A Randomized Clinical Trial

**DOI:** 10.3390/nu12051481

**Published:** 2020-05-20

**Authors:** Mireille Castanet, Christos Costalos, Nadja Haiden, Jean-Michel Hascoet, Bernard Berger, Norbert Sprenger, Dominik Grathwohl, Harald Brüssow, Nanda De Groot, Philippe Steenhout, Sophie Pecquet, Jalil Benyacoub, Jean-Charles Picaud

**Affiliations:** 1 CIC INSERM U1404, Department of Pediatric, Rouen University Hospital Charles Nicolle, 76031 Rouen, France; mireille.castanet@chu-rouen.fr; 2 Department of Neonatology, Alexandra Regional General Hospital, 11528 Athens, Greece; ccostalos@yahoo.gr; 3 Department of Clinical Pharmacology, Medical University of Vienna, Währinger Gürtel 18–20, 1090 Vienna, Austria; nadja.haiden@meduniwien.ac.at; 4 Department of Neonatology, Maternite Regionale, CHRU Nancy, 54035 Nancy, France; j.hascoet@chru-nancy.fr; 5 Nestlé Research, Société des Produits Nestlé S.A., 1000 Lausanne, Switzerland; bernard.berger@rdls.nestle.com (B.B.); norbert.sprenger@rdls.nestle.com (N.S.); dominik.grathwohl@rdls.nestle.com (D.G.); haraldbruessow@yahoo.com (H.B.); 6 SBU Nutrition, Nestlé, 1800 Vevey, Switzerland; ndegroot70@hotmail.com (N.D.G.); sophiepecquet@outlook.com (S.P.); 7 Nestlé Health Science, 1066 Epalinges, Switzerland; psteenhout@me.com; 8 Department of Neonatology, University Hospital Croix Rousse, Hospices Civils de Lyon, 69317 Lyon France; jean-charles.picaud@chu-lyon.fr; 9 INSERM U1060, INRA U1397, Claude Bernard University Lyon 1, Lyon, 69100 Villeurbanne, France

**Keywords:** early gut maturation, infant microbiota, probiotics, prebiotics

## Abstract

Background: Post-natal gut maturation in infants interrelates maturation of the morphology, digestive, and immunological functions and gut microbiota development. Here, we explored both microbiota development and markers of gut barrier and maturation in healthy term infants during their early life to assess the interconnection of gut functions during different infant formulae regimes. Methods: A total of 203 infants were enrolled in this randomized double-blind controlled trial including a breastfed reference group. Infants were fed starter formulae for the first four weeks of life, supplemented with different combination of nutrients (lactoferrin, probiotics (*Bifidobacterium animal* subsp. *Lactis*) and prebiotics (Bovine Milk-derived Oligosaccharides—BMOS)) and subsequently fed the control formula up to eight weeks of life. Stool microbiota profiles and biomarkers of early gut maturation, calprotectin (primary outcome), elastase, α-1 antitrypsin (AAT) and neopterin were measured in feces at one, two, four, and eight weeks. Results: Infants fed formula containing BMOS had lower mean calprotectin levels over the first two to four weeks compared to the other formula groups. Elastase and AAT levels were closer to levels observed in breastfed infants. No differences were observed for neopterin. Global differences between the bacterial communities of all groups were assessed by constrained multivariate analysis with hypothesis testing. The canonical correspondence analysis (CCA) at genus level showed overlap between microbiota profiles at one and four weeks of age in the BMOS supplemented formula group with the breastfed reference, dominated by bifidobacteria. Microbiota profiles of all groups at four weeks were significantly associated with the calprotectin levels at 4 (CCA, *p* = 0.018) and eight weeks of age (CCA, *p* = 0.026). Conclusion: A meaningful correlation was observed between changes in microbiota composition and gut maturation marker calprotectin. The supplementation with BMOS seems to favor gut maturation closer to that of breastfed infants.

## 1. Introduction

At birth, a still immature gut encounters a rapid colonization with microbes together with enteral diet and milk. These provide signals that drive post-natal gut maturation towards appropriate digestive and immunological functions [1,2]. To gain detailed insight in the early postnatal gut maturation processes models are often used to bridge to infant development for whom mechanistic insight is more difficult to obtain. As demonstrated in various experimental models, several aspects of the gut immune maturation depend on the interaction with a host-specific gut microbiota [3,4,5,6]. Such links between a specific microbiota and gut development are more difficult to establish in humans, although similarities are expected.

In preterm infants, Cieleborg et al. suggested a positive effect of gut microbiota on gut maturation [7]. An integrated analysis of gut maturation and gut microbiota development in breastfed and formula-fed infants provides further insight into the crosstalk between gut microbiota and the intestinal mucosa. So far, a number of studies have either investigated gut microbiota development [8,9,10,11,12,13,14,15] or gut maturation [16,17,18] in early infant life. Few studies explored those two aspects in the same infants, and such data are ultimately needed to better understand the host-microbe crosstalk. Significant health benefits have been related to human milk composition. Human milk provides numerous factors promoting gut development. Essential fatty acids intake as well as maternal and child fatty acid desaturase genotype (FADS) are also factors influencing the maturation of infant immune system [19,20,21]. Maternal milk also provides components that act directly or indirectly on the gut microbiota, either by inhibiting microbes such as lactoferrin that suppress undesired colonizers via its anti-microbial function [22,23,24,25] or by stimulating commensal bacteria. Omega-3 PUFAs are reported to impact the gut ecosystem [26,27]. Milk oligosaccharides favor the development of a bifidobacteria-dominated gut microbiota, as observed in breastfed infants [14,28,29].

In the present study we have explored in breastfed and formula fed infants both gut microbiota development and gut maturation factors in relation to different functional milk components added to formula. We hypothesized that a starter formula supplemented with different nutrients such as a prebiotic (Bovine Milk-derived Oligosaccharides, BMOS), probiotic (*Bifidobacterium animal* subsp. *lactis, B. lactis*) and glycoprotein (Lactoferrin) could support imprinting, maturation of the metabolism, and early development of gut functions closer to that of breastfed infants in association with microbiota development. Selected fecal biomarkers reflecting gut barrier and maturation were analyzed in formula-fed infants and compared to breast-fed reference group. Among the four chosen gut markers, calprotectin, a known and studied intestinal marker in newborns [30], was chosen as primary outcome and used to define the study sample size. Indeed, this calcium binding protein, produced by neutrophils, granulocytes and macrophages in the sub-mucosa, has been described as a valuable marker of intestinal inflammation, intestinal diseases, including inflammatory bowel disease (IBD) and neoplasia [31], and potential leakiness of intestinal barrier. In the first weeks of life, fecal calprotectin levels are also associated with intestinal mucosa inflammatory infiltration by neutrophils, in response to the initial bacterial colonization of the gut [32].

Furthermore, neopterin, α-1Antitrypsin (AAT) and elastase were assessed as supportive secondary outcomes. Indeed, some studies described variations on intestinal concentrations of these markers in the first 10 days of life of healthy breastfed newborns [33,34,35]. Series of publications have reported that fecal elastase levels in infant stool samples is a reliable marker of pancreatic function and overall infant metabolism development [35,36].

For the fecal AAT levels, a serine proteinase inhibitor is commonly considered as a marker of enteric protein loss. It has been shown that AAT is a relevant marker for intestinal inflammation-associated diseases [37], IBD [38] or cow’s milk allergy in infant [39]. We also investigated fecal neopterin, another marker of cell-mediated inflammation that responds to endotoxin exposure in the gut [40].

## 2. Materials and Methods

### 2.1. Objectives

The primary objective of this trial was to identify whether a standard basic formula supplemented with a combination of lactoferrin and other functional ingredients has a beneficial effect on gut maturation in full term healthy infants. Primary outcome, in order to assess primary objective, was calprotectin in stools measured at 14 days and at one month follow up.

As additional exploratory objectives, stool microbiota profiles were analyzed together with three additional protein markers besides calprotectin, namely elastase, α1-Antitrypsin (AAT) and neopterin—all measured over time. All four are markers reflecting gut barrier and maturation of infants in the first weeks of life [30,41,42,43,44,45,46,47]. Similar biomarkers’ values from the breastfed group were used as a reference.

### 2.2. Study Design

In this multi-center, randomized, double-blind, controlled trial, we included healthy term infants born in six sites from three countries: Hôpital de la Croix Rousse, Lyon, France; Maternité Régionale, Nancy, France; Hôpital Charles Nicolle, Rouen, France; Hôpital Mère Enfant CHU de Nantes, France; Alexandra Regional General Hospital, Athens, Greece; and Medical University of Vienna, Austria (Consort chart Supplementary Table S1).

### 2.3. Study Population

Healthy full-term vaginal born infants were enrolled at birth and randomized within the first 48 h. Infants enrolled in the formula groups were selected from mothers who had decided not to breastfeed, and those enrolled in the breastfed reference group were selected from mothers who had decided to exclusively breastfeed for at least two months.

The inclusion criteria were having a gestational age of 37 to 42 weeks and weighing 2500 to 4500 g at birth. Additionally, parents/caregivers had to be able to comply with the study protocol and have signed the informed consent form prior to infants being randomized. The exclusion criteria were having mothers who had taken antibiotics during the seven days preceding giving birth, being born by caesarean section, having a multiple birth sibling, having a congenital illness or malformation that could affect normal growth, having significant pre-or post-natal disease, or participating in another clinical trial.

### 2.4. Study Formulas, Randomisation, and Visits

The products were individually coded, only labelled with a number. For each center, a randomization list was generated with the help of a random number generator of a computer. Identity of the specific product was blinded to subjects, support staff and investigators. A code break envelope containing scratching cards was supplied to the study sites so that a code may be broken in the event of an emergency.

After randomization, infants received the control and test formulae until one month of age. Between one and two months of age, all infants received the same commercial starter formula. Infants in the breastfed group were not randomized and received exclusively breast milk from birth until two months.

Control formula (F) was designed to match as close as possible early maternal milk energy and proteins levels [48]. The starter formula provided 65 kcal/100 mL (in the reconstituted formula), 2.25 g protein/100 kcal (whey/casein ratio 70:30), and 5.6 g fat/100 kcal (50% milk fat). It contained carbohydrates and micronutrients in quantities that complied with the EU directive on infant formulas (2006/141/EC). Representative composition of the starter formula is shown in Supplementary Table S2. The two test formulae (FLP, FLPP) had the same nutritional composition as the control formula F (formula) but were additionally supplemented as follows: FLP (Formula Lactoferrin Probiotic) contained native bovine lactoferrin (1 g/L, DMV International, Veghel, The Netherlands) and a low dose of the probiotic *Bifidobacterium animalis* ssp *lactis* CNCM I-3446 (3.7 ± 2.1 10^4^ CFU/g powder formula); and FLPP (Formula Lactoferrin Probiotic Prebiotic) had the same composition as FLP but was additionally supplemented with a prebiotic, a mixture of bovine milk-derived oligosaccharides (BMOS) generated from whey permeate (containing galacto-oligosaccharides and other milk oligosaccharides, such as 3′-and 6′-sialyllactose) at a total oligosaccharides concentration of 4.68 ± 1 g/100 g of powder formula (6 g/L in reconstituted formula) replacing part of the lactose. Formulae were manufactured at the Nestlé Product Technology Centre, in Konolfingen, Switzerland. After the first month, the infants of the three formula groups were fed a commercially available low-protein starter formula NAN 1, SBU Nutrition, Societé des produits Nestlé, Vevey, Switzerland, with 67 kcal/100 mL energy, 1.8 g/100 kcal protein (whey/casein ratio 70:30) and 5.3 g/100 kcal fat, hereinafter “commercial starter IF”.

The study products given for the first month were packaged in individual stick packs, and individually coded with a number. Two different stick packs were prepared to cover the intervention period: one 12.2 to 12.8 g formula powder stick pack covered the first 14 days, (for a final bottle volume of 100 mL), and a second 15.8 to 16.6 g formula powder stick pack covered the period 15 to 30 days of age (for a final bottle volume of 130 mL). Study investigators and support staff as well as the infants’ parents/caregivers were all blinded to the identity of the formulae. Product from one to two months was packed in 400 g tins. Formula-fed babies were offered this formula until they were six-month old.

Visits to the study centers took place at the age of three to four days (week one visit) and at two, four, and eight weeks. At each visit, a study investigator performed clinical examinations, anthropometric measurements, and evaluated any adverse event (AE) or concomitant medication. Additionally, for infants from the study sites in Lyon and Nantes, body composition was measured using the PEA POD system (Cosmed, Brignais, France) at the one, four, and eight-week visits. Urine samples were collected at the Greek center at three to four days, and two and four weeks. Stool samples were collected at all sites at each visit. Blood samples were collected at all sites at four and eight weeks.

### 2.5. Outcome Measures

The primary outcome parameter was the fecal calprotectin mean levels (µg/g of feces) at two and four weeks of age. Secondary outcomes were fecal AAT, elastase, neopterin and sIgA concentrations, gut microbiota, fecal pH. Gut permeability was evaluated by lactulose/mannitol ratio. Additionally, anthropometrics and body composition were measured. Additionally, serum ferritin and transferrin were analyzed. Finally, digestive tolerance and AEs were recorded.

Baseline information was recorded during the first 48 h of birth, before any intake of the study formulas. This included the infants’ and parents’ demographic data and the infants’ anthropometric measurements (weight, length, and head circumference). Parents/caregivers were given diaries where they recorded digestive tolerance (stool characteristics, spitting-up, and vomiting, and infants’ behavior (crying, fussing or colic)) and any complementary feeding, AEs, or concomitant medication. Diary records were entered daily during the first week and for the three days preceding each visit thereafter.

#### 2.5.1. Stool Parameters

Stool samples were collected either by the caregivers at home within 10 h for each visit or staff at the study site. At each visit, a total of 5 to 6 g of sample was collected from each infant and aliquots of 1 g in 5-mL cryotubes stored at −20 to −80 °C. Due to the small quantities of stool obtained in the first month of life, plan of analysis was prioritized according to the following order: fecal pH, gut maturation markers and fecal IgA, and microbiota analysis. Samples from all study sites were all analyzed in the same Laboratoire de Biochimie of the Centre Hospitalier Lyon Sud. Fecal calprotectin (EK-CAL, Bühlman, Shönenbuch, Switzerland), AAT (Immundiagnostik, Bensheim, Germany), elastase (Schebo Biotech, Pancreatic Elastase 1, Giessen, Germany), neopterin (IBL, Hamburg, Germany), and sIgA (Immunotek, Zeptrometrix, Buffalo, USA) were analyzed using the ELISA methods.

Each site was provided the same type of electrode for measuring fecal pH (Metrohm^®^, Zofingen, Switzerland). pH meters were calibrated according to the manufacturer’s specifications.

To assess gut microbiota, fecal samples were suspended to a final concentration of approximately 0.5 g stool/mL in saline solution containing 10% glycerol. The mixture was homogenized and stored at −20 to −80 °C until further analysis. The 16S rRNA gene variable region V1 to V3 was PCR amplified, sequenced on Roche 454 GS-FLX-Titanium sequencer and analyzed as previously described [16]. Raw sequence data were deposited in the GenBank Short Read Archive. (Accession number: XXX)

To assess gut permeability, urine samples were collected after 6 h ingestion of a lactulose/mannitol solution [49]. Urinary concentrations of lactulose and mannitol were measured by HPLC.

#### 2.5.2. Anthropometry and Body Composition

Infants were weighed nude on electronic scales that were calibrated according to the manufacturer’s instructions every month beginning at the initiation of the study. At each site the same scale was used for all infants during all visits, and measurements to the nearest 10 g were recorded. Recumbent length was measured using standardized length boards, with two people ensuring that the body was fully extended and feet flexed. Measurements to the nearest 0.5 cm were recorded. Head circumference was taken at approximately 2.5 cm above eyebrows, directly over the largest skull circumference using non-elastic, plastic-coated measuring tapes, and measurements to the nearest 1 mm were recorded. Body composition was evaluated by measuring fat and fat-free mass at one week, and one and two months of age by a Pea Pod^®^ (displacement of air in an enclosed chamber). This was made in a subgroup consisting of all infants recruited in Lyon and Nantes.

#### 2.5.3. Iron Status

Iron status were determined at the laboratory of Pediatrics of Medical University of Vienna. Blood was collected in EDTA-containing tubes (Vacutainer^®^ Blood collection tubes, K3 EDTA, Becton Dickinson, Eysins, Switzerland). Ferritin and transferrin concentrations were determined at a centralized laboratory. Ferritin was measured by nephelometry (BECKMANN Image, Instrumentation Laboratory, Vienna, Austria), Transferrin was measured by an antigen-antibody turbidimetric method using rabbit human transferrin antiserum (Boehringer Mannheim, Indianapolis, IN, USA).

#### 2.5.4. Digestive Tolerance

Digestive tolerance was assessed based on the records kept by the parents/caregivers in the first weeks’ daily and the three-day diaries before each visit. These were frequency of spitting-up, vomiting, crying (hours/day), colic, constipation, sleep interruptions. Spitting-up was defined as bringing up small quantities of non-projectile milk after feeding, and vomiting was defined as projectile bringing up of large quantities of milk. Constipation was defined as the presence of hard stool or the absence of any stool for ≥48 h. Colic, (recorded as frequency/day) was assessed in infants up to two months of age and included: irritability, fussing or inconsolable crying that start and stop without obvious cause; episodes lasting ≥3 h per day and occurring ≥3 days per week for ≥1 week; but without failing to thrive.

#### 2.5.5. Adverse Events

In addition to AEs recorded in diaries, investigators interrogated parents/caregivers specifically regarding the occurrence of diarrhea, constipation, respiratory symptoms, fever, ear infection, skin rash, and antibiotic intake. Diarrhea was defined as three or more loose or watery stools in 24 h, and a diarrheal episode was considered to have ended once infants had two consecutive stools that were not watery or they had no stool for 24 h. Fever was defined as a temperature above 38 °C–38.5 °C at least once during a 24-h period. Respiratory symptoms were runny nose or chronic cough, which were recorded on a scale from 0 to 3 as follows: 0, absent; 1, mild; 2, moderate; and 3, severe. Atopic eczema was also rated on a similar scale. Investigators assessed all AEs for seriousness and relation to the study products. AEs were coded using the World Health Organization Adverse Drug Reaction Terminology (WHO ART).

### 2.6. Statistical Analysis

Sample size calculation was based on a previous study reporting a 35% lower fecal calprotectin concentration in preterm infants who consumed *B. lactis* compared with control infants who did not [48]. Based on this study, we derived a population standard deviation (SD) of 26%. We considered a 23% difference in calprotectin concentration between groups to be clinically significant, therefore at a α = 0.05/4 and β = 80%, 30 infants had to be enrolled in each formula group. Accounting for a 25% drop-out rate, 40 infants per formula group were required. More breast-fed infants (*n* = 66) were planned to be enrolled.

The per protocol (PP) population excluded formula-fed infants who did not adhere to exclusive feeding of their assigned regimen during the first two months of age, and breastfed infants who received less than two months of exclusive breast milk. Non-exclusive feeding was defined as intake of more than one bottle of a non-assigned formula during the first month or, between the first and second months, intake of a non-assigned formula either for more than two consecutive days or for more than a total of three days. Additionally, infants were excluded from the PP population of both the breastfed and formula groups if they had any intake of antacids or antibiotics before two months of age or if they had an intake of more than four teaspoons (approximately 20 g) of complementary food (any cereal, milk-containing cereal, cereal drink, fruit, meat, fish, eggs and other protein-rich foods, vegetable, or any other foods intended for babies). Infants who had any serious AEs (SAEs) were also excluded from the PP population.

The intention-to-treat (ITT) population included all infants in the formula groups who had any intake of the study formulas and all enrolled breastfed infants.

Data were summarized as mean ±SD (or 95% confidence interval [CI]) or median and interquartile range (IQR).

Weight, length, and head circumference were compared with WHO standards. Anthropometric data were also analyzed using a mixed model, with anthropometric data at birth, sex, treatment, visit, sex*visit interaction, and treatment*visit interaction as fixed effects and subjects as random effects. Likelihood ratio test was used to compare between groups.

Mean daily frequency of spitting up, vomiting, colic, and sleep interruptions and mean daily number of hours of crying and lag of sleep, were analyzed by Poisson regression with days of observation introduced as an offset. Serious and non-serious AEs were analyzed using the chi square test.

Calprotectin was approximately log-normally distributed, the measurements at week two and week four were averaged on the log-scale and then back transformed by applying the exponential function (the back transformed means are the geometric means). Aim of the statistical analysis was to identify which formula group is the closest to the breastfed group and which active ingredients make this happen. The primary hypothesis test was the comparison of the group which is most close to breastfeeding with the basic-starter formula without active ingredients.

Treatment differences of log-normally distributed variables can be interpreted in good approximation as percentage changes with respect to some grand mean: d log(x) = dx/x.

Similarly, geometric mean values of other biochemical markers (including iron and IGF-1) were calculated from all the measurements taken at the various time points, i.e., neopterin and elastase from the 3–4-day, and 1, 2, and 4-week visits; fecal AAT from the 3–4-day and 1, 2, and 8-week visits; IgA from the 4 and 8-week visits, and the lactulose/mannitol ratio from the 3–4-day and 2 and 4-week visits. T-test was used to compare between groups. In case of outliers, the Wilcoxon rank-sum test was performed, and the treatment difference was quantified using the Hodges-Lehmann estimator with 95% CI.

Z-scores for biochemical markers were calculated relative to the breastfed infants using the mean and SD values from the breast-fed group.

Statistical analyses were performed using SAS (SAS Institute Inc, Cary, NC, USA). For the analysis of the microbiota 16S rRNA genes pyrosequencing data, the diversity analyses and correlations with clinical data were performed with the website Calypso. [50]

### 2.7. Ethics

The study was conducted in accordance with the Declaration of Helsinki and its subsequent amendments. It conformed to the International Conference on Harmonization guidelines for Good Clinical Practices and adhered to the applicable regulatory and legal requirements. The study was approved by the ethics committees of each institution: Lyon CPP Sud Est IV Ethics Committee and the French Agency for the Safety of Health Products (AFSSAPS) in France; Alexandra Hospital Scientific Council in Greece; and the Vienna Ethics Committee and the local regulatory authority (AGES) in Austria. The clinical trial has been registered on ClinicalTrials.gov on September 10, 2009 (NCT00984230).

## 3. Results

### 3.1. Study Population

**Figure 1:**
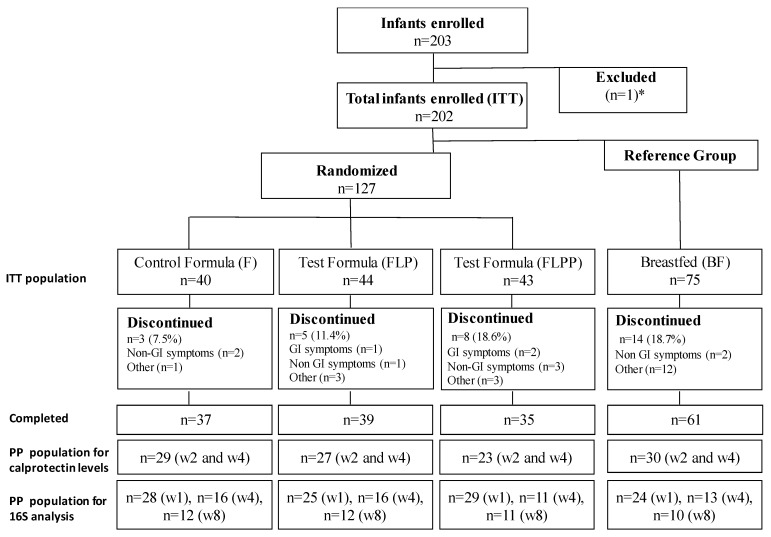
Flowchart of infants participating in the study (Intention To Treat, ITT) and 16S (Per Protocol, PP) analysis. * 203 infants were enrolled; one was excluded due to a randomization error. The total number of infants enrolled was 202. GI is gastrointestinal symptoms, w2 and w4 means week 2 and week 4.

Overall, 203 healthy term infants were enrolled; 127 infants were randomized to formula; 75 mothers decided to breastfeed; and one mother decided to formula feed, but was not randomized. Recruitment lasted from September 2009 to December 2010 in three European countries: France, Austria, and Greece. The infants were randomized to one of three formula-fed groups: group F (*n* = 40), group FLP (*n* = 44), and group FLPP (*n* = 43), constituting the ITT population. The total number of infants who completed the study to two months was 162 (F: *n* = 37; FLP: *n* = 39; FLPP: *n* = 35; BF: *n* = 61). The 16S rRNA microbiota analysis was performed on the PP population (Figure 1).

All infants were singletons and born vaginally. At birth the groups are well comparable with respect to body weight, length, head circumference, gender and time from birth to enrolment (1.5 ± 0.7 days) (Table 1).

### 3.2. Gut Barrier Function Maturation

Calprotectin levels were within the physiological ranges when compared to the BF group in the three formula groups. We observed a wide range of fecal calprotectin values in breastfed infants (Table 2). The primary hypothesis test was FLP group vs. F group. The geometric means were 346 µg/g and 321 µg/g in FLP group and F group, respectively. Closest to the BF group (386 µg/g) was the FLP group (346 µg/g) (Table 2).

**Figure 2:**
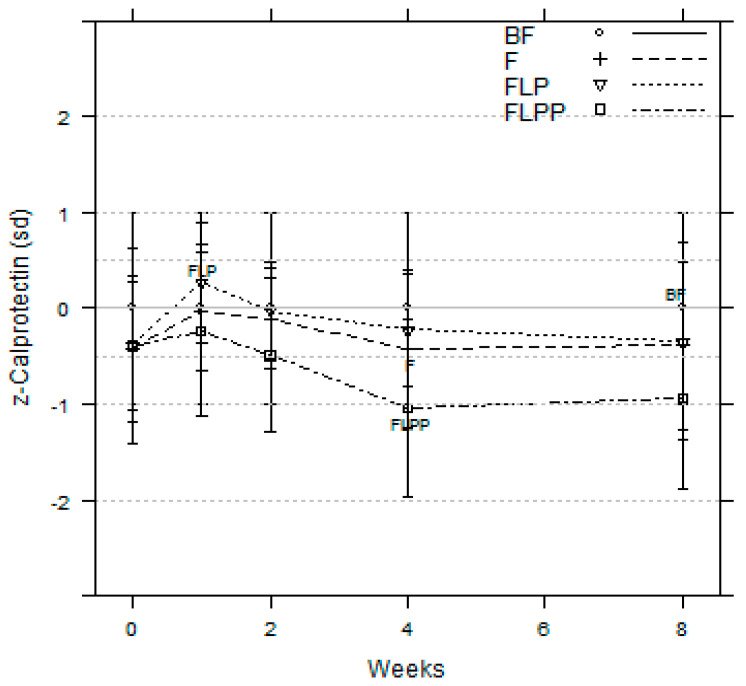
Fecal calprotectin z-scores compared to breastfed group set as zero line (PP population).

FLPP group showed lower calprotectin levels all along the intervention (Figure 2). At two, four, and eight weeks, significant differences appeared between FLPP and BF groups, with respective *p* values of 0.041, 0.003, and 0.012. When compared to the breastfed reference, z-scores showed that the FLPP group is within – 1 standard deviation while FLP and F were within −0.5 SD (Figure 2). During the intervention calprotectin levels were also significantly different between FLP and FLPP groups at week 2 (*p* = 0.044) and 4 (*p* = 0.002). A significant difference between FLPP and F groups was only observed at week 4 (*p* = 0.031) (Figure 2).

The overall analysis of fecal calprotectin and other parameters to assess the early development of the gut over the trial period are presented as absolute values at week 4 (Table 3)

Levels of AAT were significantly lower in the FLPP group than F and FLP groups. Z-scores values were within the ± 0.5 SD (data not shown).

No increase in fecal neopterin concentrations was observed along the intervention period in all groups. However, during the first month of life, neopterin levels were significantly lower in breastfed infants than in formula-fed infants. During and after the intervention, there were no significant differences between the three formula fed groups. Z-scores values were within the ± 0.5 SD at week 8 (data not shown).

Concentrations of fecal elastase in both FLPP and BF were not significantly different from birth up to four weeks during the intervention period, while the concentrations were significantly higher in the F and FLP groups when compared to BF. The effect was no longer maintained after the one-month feeding with commercial starter formula and all intervention groups showed similar elastase levels at eight weeks (data not shown).

No difference in IgA level was seen between the different formula groups while the breastfed group showed four-times higher stool IgA levels at four weeks of age.

No significant difference in gut permeability, assessed by urinary lactulose and mannitol excretion, was observed between the different feeding groups.

### 3.3. Stool Microbiota

Stool microbiota analysis by 16S RNA gene sequencing.

**Figure 3:**
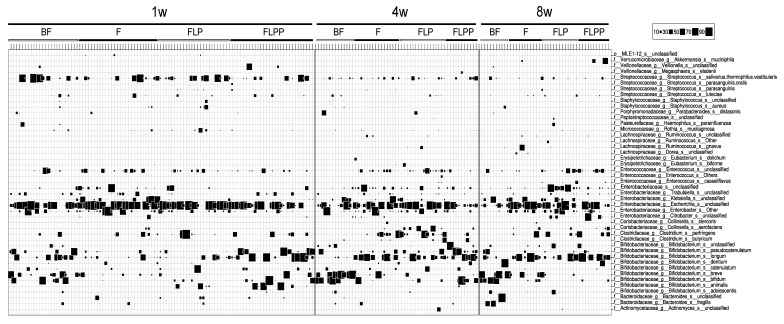
Gut microbiota composition in infants from the four nutritional intervention groups (PP data set). Determination by 16S rRNA gene sequence analysis: bubble plot of bacteria determined at species level (identified at the right ordinate) for the intervention groups and time points specified on the top abscissa. The proportion of the specified bacterium in the specified sample is indicated by the size of the square (key at top right of the figure).

At one, four, and eight weeks of age stool samples from 106, 56 and 45 infants, respectively, were available for microbiota analysis. The microbiota composition of each sample is displayed in Figure 3. The results of statistical tests when comparing the feeding groups are reported in Table 4. Each nutritional group was at a given time point represented by at least 10 infants (for details, see Figure 1).

At one week of age, few bacterial taxa dominated the stool composition: *Escherichia*, *Bifidobacterium*, *Streptococcus*, *Enterobacter* and *Enterococcus* (in this decreasing order). Infants in the FLPP group showed with a median abundance of 54% a significantly higher *Bifidobacterium* level than the F and FLP groups—3% and 5%, respectively. On the contrary, this abundance was not significantly different from the breastfed physiological reference group (28%). Additionally, the breastfed group displayed a significantly higher *Bifidobacterium* level than the F and FLP formula groups. *Staphylococcus* (mainly *S. aureus*) were more abundant (0.1%) in breastfed infants than in the formulas fed groups, whereas *Klebsiella* and unclassified *Enterobacteriaceae* counts were significantly higher in the F group, respectively—0.1% and 0.2% compared to 0% in the other groups. *Bifidobacterium longum*, *B. pseudocatenulatum*, and *B. bifidum* were the dominant bifidobacteria identified in both FLPP and B groups (Figure 3).

At four weeks of age, and thus at the end of the differential nutritional intervention, *Bifidobacterium* clearly dominated in both FLPP group (77%) and breastfed infants (81%), but not in control (F) (34%) and FLP (16%) groups (Table 2). The species contributing to the increase in bifidobacteria was mainly *B. breve* in the BF group and *B. longum* in the formula groups (Figure 3). Groups F and FLP showed significantly higher levels of *Clostridium perfringens* and *Enterococcus* (unclassified at species level) than groups BF and FLPP from which they were absent. With 0.2%, the breastfed infants still harbored a significantly overall higher abundance of *Staphylococcus* (mainly *S. aureus* or closely related species).

**Figure 4:**
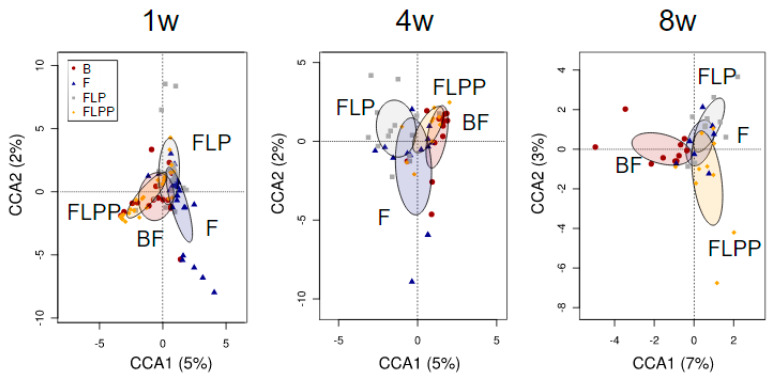
Canonical correspondence analysis at genus level for the nutritional intervention groups (PP data set). Ellipses indicates the 95% confidence intervals around the centroids. BF, breast fed; F, control formula; FLP, formula lactoferrin probiotic; FLPP, formula lactoferrin probiotic prebiotic.

After a one-month wash-out period on the commercial starter IF, i.e., at the eight weeks’ time point, no significant difference in taxa abundance was detected between formula groups. Breast-fed infants showed a continued dominance of bifidobacteria at a median value of 70%, while this taxon abundance decreased to less than 30% in all formula groups (Figure 4). The strong bifidogenic effect observed in the FLPP group compared to the FLP and F groups was thus only maintained during the intervention period.

Diversity analyses over time. At no time point the four nutritional groups significantly differed for community (alpha) diversity (data not shown). The global differences between the bacterial communities of the formula fed groups and the breastfed physiological reference group were assessed by constrained multivariate analysis with hypothesis testing. The canonical correspondence analysis (CCA) at genus level separated the BF and FLPP groups from the F and FLP groups, with the data points for the FLPP formula group overlapping with the breastfed reference group at one (*p* = 0.001) and four weeks (*p* = 0.043) of age (Figure 4, 1w and 4w). In contrast, at eight weeks of age after washout period with the commercial starter IF, all three formula groups were separated from the breastfed group (*p* = 0.026, Figure 4, 8w).

The 16S rRNA gene sequencing dataset showed that *B. animalis* spp *lactis*, to which the added probiotic belongs, was mainly detected in samples of the FLP and FLPP supplemented intervention groups (Table 4) where they made only a minor contribution to the total *Bifidobacterium* population (Figure 3).

### 3.4. Association between Microbiota Profiles and Calprotectin Levels

To investigate the role of the microbiota in the gut barrier maturation, we tested at each time point the association of the microbiota composition (genus level) with concomitant and subsequent calprotectin levels (defined as primary measure). The analysis considered the BF group only, the formulae groups only or all groups together. The microbiota profiles of the all groups dataset at four weeks were significantly associated with the calprotectin levels at four weeks (CCA, *p* = 0.018) and eight weeks of age (CCA, *p* = 0.026). In contrast, no significant association of fecal elastase levels with microbiota composition was observed. In univariate analysis, no clear association was observed between the genus abundances and calprotectin levels.

### 3.5. Fecal pH

At one and four weeks, stool pH in the FLPP group was significantly lower compared to the other groups. This difference disappeared after the intervention period. Breastfed infants displayed a significantly lower stool pH than all formula-fed infants except FLPP infants at one week of age (Supplementary Figure S1).

### 3.6. Growth (Anthropometry and Body Composition)

**Figure 5:**
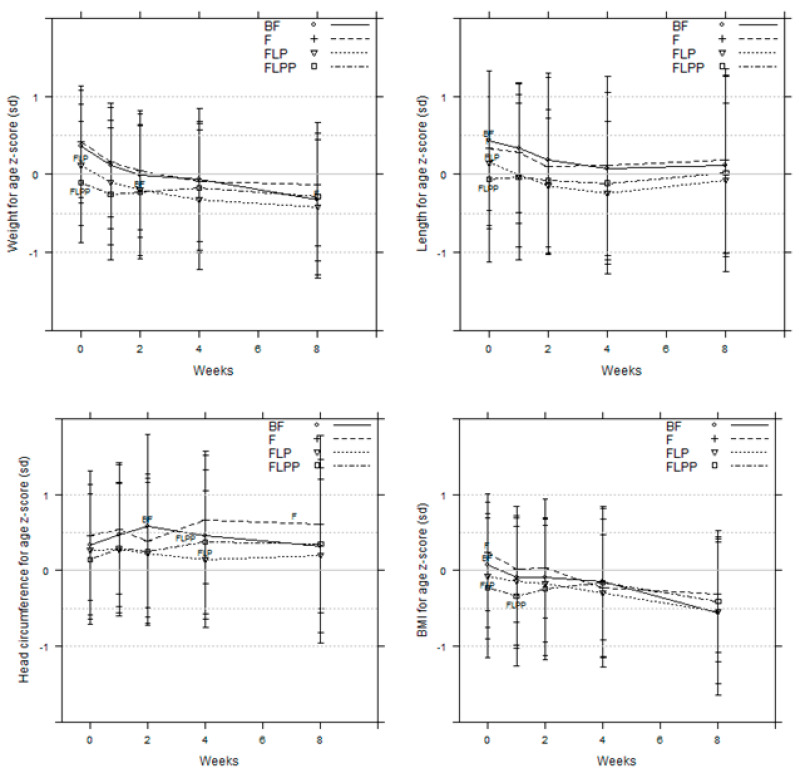
Anthropometric data. z-scores for body weight, length, head circumference, and BMI (PP data set).

No significant difference in growth was observed (Figure 5). All anthropometric characteristics closely followed the WHO standard curves. At the end of the intervention, four weeks’ time point, the body composition of the formula fed infants showed a lower percent of fat-free mass compared to breastfed infants, but this difference was not significant (data not shown).

### 3.7. Iron Status

Lactoferrin supplementation for one month after birth had no impact on the iron status when compared to F or BF groups with respect to serum iron, ferritin, transferrin, and soluble transferrin receptor levels (data not shown).

### 3.8. Digestive Tolerance

During the intervention period, no significant difference was observed between the different feeding groups for spitting-up and vomiting frequencies, infant’s crying time and colic frequency. Breastfed infants showed a significantly higher number of sleep interruptions than the formula-fed infants (data not shown). The FLPP group showed less hard and formed stools and more soft stools compared to F and FLP groups (Supplementary Figure S2). These differences only lasted during the intervention period.

### 3.9. Adverse Events/Morbidity

Adverse events during the first two months of life consisted mainly of gastrointestinal and respiratory diseases. No difference was seen between all feeding groups (data not shown).

## 4. Discussion

In this study, we investigated early gut maturation in healthy term infants with different feeding regimens. The supplementation including the mixture of bovine milk derived oligosaccharides (BMOS) globally favors a gut maturation compared to the control formula, getting closer to that observed in breastfed infants for some, but not all, markers used here. We observed a meaningful correlation between changes in microbiota composition and gut maturation indicators, such as calprotectin. All formulae were well-tolerated and assured safe growth of the infants. The novelty of this study was to follow four gut maturation markers all together in healthy term infants during their early life, and to hypothesize on interaction with initial microbiota colonization, according to their infant formula regimen.

Previous nutritional interventions with a synbiotic of BMOS and the same B. lactis strain led to a strong increase of the bifidobacterial population compared to control infants, further supporting the bifidogenic effect of BMOS [14,51]. In both studies, B. lactis was increased in the probiotic-supplemented groups, but it remained a minor constituent of the total fecal *Bifidobacterium* composition. From this observation we concluded that the prebiotic component of the synbiotic mixture had a greater effect than the probiotic component on the observed gut microbiota shift. In the present study, the BMOS was the only component which differed between the FLPP and FLP supplements and was thus likely the driver for the observed bifidogenic effect. We used a low probiotic concentration (4 × 10^4^ cfu/g as compared to regular dose of 10^7^ cfu/g), which appeared to have no effects on gut markers as well as microbiota composition over the control. Beyond its bifidogenic effect, BMOS supplementation modulated the global microbiota composition towards the BF reference microbiota as early as one week after the beginning of the intervention. The effect did not persist after cessation of the differential interventions.

By analyzing our primary outcome, we first found a clear difference on calprotectin levels between formula fed and breastfed infants as often observed [52,53,54,55]. For all formula fed infants, including the control group, the ranges of fecal calprotectin concentrations varied from 90 and 600 µg/g, while the upper range observed in breastfed infants reached almost two-fold higher levels (1044 µg/g). This could be ascribed to immune stimulating factors in breastmilk or transmission of calprotectin during suckling.

Moreover, the constant ranges of calprotectin observed in the formula fed infants might be due to the fixed standard composition of the formula used, while the composition of breast milks likely differs between individual lactating mothers and overtime [56,57,58]. To date, it is not clear as to why breastfed infants showed higher fecal calprotectin. Breastmilk is possibly a source of calprotectin that escapes digestion in the infant. Alternatively, different breastmilk components such as cytokines may stimulate mucosal calprotectin release in breastfed infants [54,55]. However, mean calprotectin levels were higher in the control and FLP groups compared to the FLPP group, which might be linked to differences in early microbiome colonization. In fact, in our study, higher fecal calprotectin concentrations correlated with the Staphylococcus, Escherichia, and Klebsiella abundance in the gut microbiota. These bacteria are common pioneer gut colonizers in formula fed infants. Since they also represent bacterial groups containing facultative pathogens as members, an increased calprotectin secretion by neutrophils might be induced. Similar to our results, calprotectin increase was not associated with bifidobacteria colonization but with Escherichia, in a broad age range Dutch population [59].

The second parameter of interest during this study appeared to be the elastase. It is reported that fecal elastase levels in infant stool samples is a reliable marker of pancreatic function and overall infant metabolism development [35,36]. Fecal elastase levels above 200 µg/g of stool indicate normal pancreatic function and normal overall infant metabolism development [60,61]. All infants from this trial presented fecal elastase concentrations above this physiological threshold. Within the first four weeks of their life, infants fed with the FLPP formula only presented levels of fecal elastase comparable to those of breastfed, both being lower than the other formula fed groups. This suggests that the BMOS supplementation from the FLPP contributes to a positive effect on metabolic homeostasis, possibly through the microbiome. This is supported by the observation that the effect on stool elastase was only observed during the FLPP feeding period and was lost thereafter.

In the present study, similarly to what was observed for the elastase, AAT levels in the group fed FLPP were closer to the ones observed in the breastfed group. Nevertheless, AAT levels remained within normal range over the study period for all groups, confirming the absence of detrimental inflammation and intestinal barrier damage.

Finally, fecal neopterin, did not differ between the various formula-fed groups. This indicates an absence of pathogenic infections during the gut colonization and supports the ongoing establishment of a well-balanced microbiome [40].

A limitation of the study is that we mainly investigated biological parameters to evaluate gut maturation, and safety parameters. From that perspective, we observed that all formulae assured age-appropriate growth of the infants. No difference was seen between the formula fed groups and the breastfed group for anthropometry and body composition. All infants grew according to the WHO growth curves. Furthermore, there was no difference between the different feeding groups. Moreover, the relatively short duration of evaluation is also a limitation of the study. Indeed, the study focused on very early effects of feeding regimen on gut maturation over the first two months of life. Therefore, we were not able to investigate further potential beneficial health effects of the intervention in the population. However, some products had a significant effect on gut microbiota, favoring a pattern close to BF infants, which could have positive long-term effects. Another limitation of the study is a large loss of infants to follow-up at weeks 4 and 8. This is not so unusual in such clinical trial involving newborns and it is important to notice that these drop-outs were not due to intolerance to formula. Overall, these promising data would need to be further confirmed in a larger scale study.

An interesting observation was that, with respect to stool consistency and stool pH, the FLPP formula kept an intermediate position between formula-and breast-fed infants. FLPP and BF groups are both dominated by bifidobacteria. Since their metabolic end products of hexose catabolism via the bifidobacteria shunt is a mixture of lactic and acetic acid [62] the observed effect on pH could be explained. Acetic acid is a member of the short chain fatty acids (SCFA) that have important physiological effects which go well beyond the gut potentially defining a gut-brain, and a gut-skin axis [63]. This drop in stool pH was consistently observed in previous studies that evaluated BMOS in infant nutrition [14,64]. In the future, it will be important to extend the stool analysis beyond stool pH measurement to a chemical profiling of the stool with respect to SCFA metabolites.

## 5. Conclusions

This innovative intervention trial provides important insight on formula supplementation for early life gut maturation by integrating, from birth, gut biomarkers evolution and development of the microbiome. In particular, the supplementation including BMOS favors gut maturation indicators including reduced AAT, elastase and calprotectin compared to control formula fed infants. The reasons and physiological significance of the highest calprotectin levels in stool of breastfed infants warrants further investigation. Our data show important correlations between changes in microbiota composition and gut maturation indicators, such as calprotectin, and thus provide an integrative insight into neonatal gut physiology.

## Figures and Tables

**Table 1:** Baseline characteristics of the 202 healthy term infants included in the study.

	BF (*n* = 75)	F (*n* = 40)	FLP (*n* = 44)	FLPP (*n* = 43)
**Mothers**				
Weight, kg (range)	58.0 (52; 65)	61.0 (54.2; 69.2)	64 (55.5; 69.5)	61.0 (55.0; 71.2)
Height, cm (range)	166 (160; 169)	165 (162; 170)	165 (162; 170)	164 (160; 170)
Age, years (range)	30.0 (27.0; 33.0)	30.0 (25.8; 33.2)	28.5 (24.2; 30.0)	28.5 (24.2; 33.8)
Former smoker, %	11	20	27	14
**Infants**				
Male gender, %	52	57.5	38.6	48.8
Age at enrolment, days	1.45 ± 0.76	1.45 ± 0.71	1.52 ± 0.63	1.4 ± 073
Weight, kg	3.45 ± 0.37	3.49 ± 0.36	3.33 ± 0.43	3.24 ± 0.38
Crown-heel length, cm	50.6 ± 1.7	50.5 ± 1.8	50.0 ± 1.6	49.6 ± 2.0
Head circumference, cm	34.7 ± 1.2	34.9 ± 1.1	34.6 ± 1.0	34.5 ± 1.1
Body mass index, kg/m^2^	13.5 ± 1.1	13.7 ± 1.0	13.3 ± 1.0	13.1 ± 1.2

B, breast fed; F, control formula; FLP, formula lactoferrin probiotic; FLPP, formula lactoferrin probiotic prebiotic.

**Table 2:** Fecal Calprotectin concentrations (mean at 2 and 4 weeks). PP data set.

	BF (*n* = 30)	F (*n* = 29)	FLP (*n* = 27)	FLPP (*n* = 24)
Mean (range) faecal calprotectin concentrations (µg/g of faeces)	386 (143, 1044)	321 (174, 594)	346 (201, 596)	205 (90, 470)
*p*-value (versus breastfed infants)		0.338	0.686	0.014

**Table 3:** Gut maturation parameters at week 4.

	BF	F	FLP	FLPP
Calprotectin µg/g (range)	444 (158;1251)	289 (123; 678)	352 (193; 641)	152 (59; 396) † *p* = 0.003 ∆ *p* = 0.031 □ *p* = 0.002
Elastase µg/g (range)	725 (485; 1085)	1084 (532; 2208) † *p* = 0.010	1129 (754; 1689) † *p* < 0.001	631 (301; 1324) ∆ *p* = 0.002 □ *p* = 0.010
Alpha 1-AT mg/g (range)	0.175 (0.084; 0.363)	0.273 (0.169; 0.442) † *p* = 0.011	0.239 (0.165; 0.345)	0.133 (0.062; 0.288) ∆ *p* = 0.002 □ *p* = 0.010
Neopterin pmol/g (range)	597 (367; 971)	849 (514; 1403) † *p* = 0.048	973 (557; 1699) † *p* = 0.008	948 (683; 1317) † *p* = 0.002
IgA µg/g (range)	619 (283; 1354)	78 (20; 309) † *p* < 0.001	132 (75; 234) † *p* < 0.001	117( 40; 340) † *p* < 0.001
Permeability Index (range)	0.774 (0.455; 1.316)	0.635 (0.318; 1.267)	0.647 (0.292; 1.435)	0.783 (0.459; 1.334)

†: significantly different from BF, ∆: significantly different from F, □: significantly different from FLP. PP data set.

**Table 4:** Statistical analyses of the differences of median between groups at genus and species levels.

Time	Genus/Species	B		F		FLP		FLPP		Significance of Differences					
		Median	Mean	Median	Mean	Median	Mean	Median	Mean	ALL	B vs. F	B vs. FLP	B vs. FLPP	F vs. FLPP	FLP vs. FLPP
**1w**	Bifidobacterium	28.3	36.7	3.0	7.2	5.3	14.1	54.2	46.7	***	***	*	NS	***	**
	Staphylococcus	0.1	0.5	0.0	0.2	0.0	1.8	0.0	0.0	***	*	NS	***	NS	*
	Klebsiella	0.0	0.3	0.1	9.3	0.0	0.7	0.0	0.1	***	**	NS	NS	**	NS
	unclassified Enterobacteriaceae	0.0	0.8	0.2	5.1	0.0	3.1	0.0	0.9	*	**	NS	NS	**	NS
	unclassified Klebsiella	0.0	0.3	0.1	9.3	0.0	0.7	0.0	0.1	***	**	NS	NS	**	NS
	Staphylococcus aureus	0.0	0.3	0.0	0.2	0.0	1.2	0.0	0.0	***	NS	NS	***	NS	*
	Bifidobacterium animalis	0.0	0.0	0.0	0.0	0.0	3.3	0.0	7.4	**	NS	*	*	**	NS
	Streptococcus parasanguinis	0.0	0.0	0.0	0.1	0.0	0.4	0.0	0.3	**	NS	**	*	NS	NS
4w	Enterococcus	0.0	0.1	1.6	3.7	3.4	6.3	0.5	1.7	***	***	***	**	NS	NS
	Bifidobacterium	81.0	61.7	34.5	32.5	16.0	23.8	77.1	61.5	**	*	**	NS	**	**
	Staphylococcus	0.2	0.6	0.0	0.1	0.0	0.0	0.0	0.1	**	NS	**	NS	NS	NS
	Clostridium	0.0	0.4	1.0	9.8	2.5	14.4	0.0	4.0	*	NS	**	NS	NS	*
	unclassified Enterococcus	0.0	0.0	1.6	3.7	2.4	5.6	0.5	1.7	***	***	***	**	NS	NS
	Bifidobacterium breve	35.3	33.7	0.0	5.0	0.0	1.1	0.0	0.2	**	*	**	*	NS	NS
	Clostridium perfringens	0.0	0.1	0.6	9.3	2.0	12.0	0.0	1.3	**	*	**	NS	NS	*
	Staphylococcus aureus	0.0	0.5	0.0	0.1	0.0	0.0	0.0	0.1	*	NS	*	NS	NS	NS

Rank test for all groups and paired comparison (PP—population). Significance: NS—non-significant; *, p-value > 0.05; **, p-value > 0.01; ***, p-value > 0.001; B, breast fed; F, control formula; FLP, formula lactoferrin probiotic; FLPP, formula lactoferrin probiotic prebiotic.

## Supplementary Materials

The following are available online at https://www.mdpi.com/2072-6643/12/5/1481/s1, Figure S1: Mean fecal pH at 4 and 8 weeks (PP data set), Figure S2: Stool consistency (ITT data set). Table S1: CONSORT 2010 checklist of information to include when reporting a randomized trial. Table S2: Representative composition of the formula.
